# A Comparison of ChatGPT and Multidisciplinary Team Meeting Treatment Recommendations in 10 Consecutive Cervical Cancer Patients

**DOI:** 10.7759/cureus.67458

**Published:** 2024-08-22

**Authors:** Florian Ebner, Andreas Hartkopf, Kristina Veselinovic, Fabienne Schochter, Wolfgang Janni, Stefan Lukac, Davut Dayan

**Affiliations:** 1 Department of Obstetrics and Gynecology, Alb-Donau Klinikum (ADK), Ehingen, DEU; 2 Department of Obstetrics and Gynecology, University of Tübingen, Tübingen, DEU; 3 Department of Obstetrics and Gynecology, University of Ulm, Ulm, DEU; 4 Department of Obstetrics and Gynecology, University Hospital of Ulm, Ulm, DEU

**Keywords:** care, tumor, advice, multidisciplinary team, treatment, cervical cancer, chatgpt

## Abstract

Background

The preparation of multidisciplinary team (MDT) meetings can be time-consuming. In addition to the clinical data being available digitally in subsystems, the preparation of more complex cases requires literature research. Several expert systems have been developed to support this process. However, the interaction with these systems has to be trained. Current development enables linguistic interaction with such artificial intelligence (AI) systems. To the best of our knowledge, these have not been tested as premedical screening tools for MDT.

Methods

This is a retrospective consecutive case series of 10 cervical cancer cases comparing the medical recommendations of the MDT and artificial intelligence (AI) on a low level (i.e., surgery, systemic treatment, and radiotherapy).

Results

The clinical cases ranged from primary diagnosis via suspected recurrence to palliative settings. The AI repeatedly stated that medical professionals need to be consulted before treatment decisions. The AI answers ranged from no agreement to overachievement by mentioning treatment options for preexisting risk factors (such as obesity). In standard cases, the AI answer matched well with the expert recommendations. In some cases, the AI answers were contrary to our treatment recommendation.

Conclusion

The interaction with current language AIs is temptingly easy, and the replies are very understandable. Despite the AI warning regarding medical recommendations in the majority of our cases, there was a good match with the MDT recommendations. However, in some cases, the medical evidence behind the answers was missing or in the worst case fictional. In our case series, the AI did not meet the requirements to support a clinical MDT meeting by prescreening the therapeutic options. However, it did exceed the expectations regarding the risk factors of the patients.

## Introduction

In our clinical context, registrars prepare the patient history for multidisciplinary team (MDT) meetings. This also includes a proposal for further treatments, which will then be refined by the MDT participants. Contrary to repeating standard situations, incomplete files/information or difficult clinical situations remain time-consuming. To minimize this time, more experienced registrars prescreen the MDT forms.

In science fiction films, however, the medical doctor is often an artificial intelligence (AI) or is supported by one (i.e., The Doctor (Star Trek: Voyager), Dr. Julian Bashir (Star Trek: Deep Space Nine), Dr. Will Caster (Transcendence), Dr. Leo Quintum (All-Star Superman)). Up until now, communication with such an expert system has been limited to a range of subjects. Although highly specialized, the interaction is based on keywords and search algorithms. However, with an increasing clinical workload, a more natural interaction with support systems is essential. Recent advances in artificial intelligence programming enable users to enter a normal linguistic request and receive an equally verbalized answer. Currently, a few systems are freely available to experiment with (XLNet, Transformer-XL, Bidirectional Encoder Representations from Transformers (BERT) by Google, and Conditional Transformer Language Model (CTRL) by Salesforce) [1].

To estimate the usability of an AI, we compared the final MDT recommendations with AI replies. Ideally, we were hoping to find a good correlation and, if applicable, implement such a support system in the preparation of MDT meetings. This could include researching appropriate guidelines, literature, or other scientific evidence.

## Materials and methods

In March 2023, this qualitative study was initiated after consulting the ethics committee. In the multidisciplinary team meeting, treatment recommendations for 10 consecutive patients with cervical cancer were collected. The endpoint was set at 10 cases to complete in a timely manner and within one software version of the AI. The anonymous patient history was entered into ChatGPT (released March 14) (OpenAI, San Francisco, CA) in the same format as the patient introduction to the MDT meeting. This study was previously presented as a meeting abstract at the 2024 Bavarian Obstetrics and Gynecology Conference (BGGF) on June 6 and at the Global Academy of Women Cancer on July 5.

Study population

For this retrospective proof of concept qualitative study, the MDT recommendations were taken to simulate a real-world clinical situation. Starting with the third MDT meeting in January 2023, recommendations were included. The 10th recommendation was taken from the February 3 MDT meeting. From a German IP address, researcher #1 conducted the ChatGPT query in a timely manner to the individual MDT meeting, while researcher #2 conducted the query over a weekend once the 10 cases were discussed at the MDT meeting. With the regular protocol of the institution, patients consent to the use of their anonymized data. The inclusion criterion was a confirmed diagnosis of cervical cancer. Age and patient history were extracted from the MDT form. All patients at our institution gave written consent to evaluate available data for scientific activities prior to the treatment. The data was provided anonymized to the investigators, so there was no possibility for the investigators to identify the patients. The extracted information was entered into the ChatGPT bot. The answers were then copied and classified accordingly.

The clinical presentation of the patients included adjuvant cases with histological confirmation of cancer in cone biopsies, patients with histologically confirmed positive lymph nodes in the primary surgery, palliative patients with and without symptoms, and suspected resectable and non-resectable recurrences. Patients were between 37 and 68 years old and presented a variety of risk factors.

Artificial intelligence

The publicly available artificial intelligence chatbot ChatGPT is a transformer-based language model. Unlike search engines, it can generate human-like text and has been trained on data up to 2021, with limited knowledge of events thereafter. The question can be entered via a website, and ChatGPT captures the context and relationship of the words in the question. The training data is based on diverse sources of text including websites, articles, and books up until 2021 [2]. It uses the most likely answer based on previous trained patterns in the data. The used GPT Model was 3.5, with ChatGPT February 13 version.

MDT criteria

In certified centers, the recommended treatment for each patient is discussed in an MDT meeting attended by experts responsible for the treatment (gynecologic oncologists, radiologists, radiation therapists, and pathologists). Treatment modalities are surgery, radiotherapy, chemotherapy, and antibody treatment or clinical trial participation. The order of the treatments can vary according to age, cancer stage, and risk factors. The MDT recommendations were used as a standard reference baseline for ChatGPT answers. In brief, the modalities of the MDT recommendation were checked against the AI modality recommendation. The consensus was categorized into "definite" consensus, "maybe" consensus, and "appropriate" consensus. Details regarding drugs, type of surgery, or radiotherapy were not used in our study.

Model input

One entry for each patient was used similar to the patient introduction in the MDT in an open-ended format: "How should the following [x]-year-old patient be further treated? (TNM, previous history of treatments, risk factors, imaging results, pathology results, etc.)." This simulates how a resident might actually interact with ChatGPT. To avoid the influence of prior answers on model output, the ChatGPT session history was deleted for each prompt. To account for response-by-response variation, each prompt was tested two times on different days.

Workflow and output scoring

**Figure 1:**
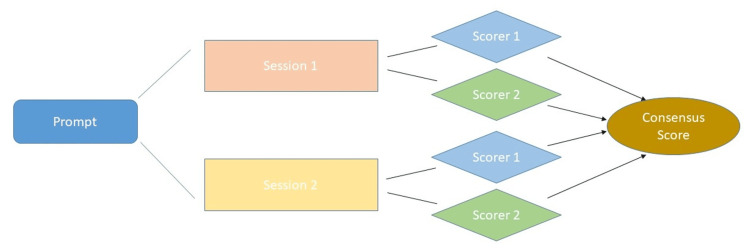
Schematic workflow of consensus score

Each prompt was put in twice and each time in a different ChatGPT session. Two scorers independently calculated an individual score for each output to confirm consensus on all output scores. A schematic of the workflow can be found in Figure 1, and scoring criteria can be found in Table 1. For example, a patient with surgery, radiotherapy, chemotherapy, and an increased body mass index (BMI) could be scored with 2+2+2+2 (requesting weight monitoring) points. The ChatGPT points were added and divided by the sum of the possible maximum points. So, the percentage provided a consensus score between ChatGPT and MDT recommendations. The recorded points of the first and second sessions were identical and also congruent between the two scorers.

**Table 1:** Answer scoring system Scoring system with MDT meeting recommendations as standard MDT: multidisciplinary team

ChatGPT did not mention/incorrect treatment modality (0)	0
ChatGPT mentioned treatment modality as a possible treatment (+/-)	1
Requesting further tests without clinical relevance (+/-)	1
Correctly requesting additional tests or results (+)	2
ChatGPT mentioned treatment modality as definite treatment (+)	2

Statistical analysis

Descriptive statistics are used for the full-text answers. Further analysis was done via chi-square testing for each treatment option (surgery, chemotherapy, and radiotherapy) and overall treatment modalities to identify a statistical difference despite the low number.

## Results

The entered data was processed within seconds and returned in a schematic answer (Appendices). The algorithm repeated the question and provided some general background information. The possible treatment options (surgery, chemotherapy, radiotherapy, and others) were then mentioned if possibly applicable. Two answers explicitly mentioned the warning that the algorithm is not a medical advisor/doctor and that treatment recommendations need to be discussed with a doctor. Every answer had a recommendation to consult a doctor before final treatment decisions. In three cases, there were comorbidities (such as adipositas, hypertension, and incomplete family planning) in the patient history. These were mentioned in all cases in the answers as additional risk factors and the possible impact on the long-term survival including treatment options (weight loss or consult a doctor for further advice).

For pT1b1 cancer (case #1), the necessity of radio-chemotherapy was overestimated compared to the MDT recommendations, or the extent of the surgery was indecisive (case #8: incomplete family planning) as a more radical surgery could have a better survival result. The answers for the International Federation of Gynecology and Obstetrics (FIGO) II cases (#2, #4, and #7) ranged from radio-chemotherapy due to vaginal bleeding (MDT: biopsy for histology) to surgery, chemotherapy, and radiotherapy (MDT: lymph node excision (LNE) before radio-chemotherapy). In patients with a tumor recurrence (#5, #6, and #10), the recommendations ranged from all treatment modes (MDT: for #5, if resectable, then surgery and if otherwise, radio-chemotherapy; for #6, positron emission tomography-computed tomography (PET-CT), and urology checkup) to biopsy of the suspected recurrence (MDT: PET-CT). Case #3 was introduced due to unclear (but histologically benign) enlarged inguinal lymph nodes, so the MDT recommended a "wait-and-see" approach, contrary to the surgical approach of the algorithm. Our concordance score ranged from 0 to 6 with an average of 1.7 and 72.5% agreement (Table 2).

**Table 2:** Patient overview Input example case #1: Question: How should the following 47-year-old patient be further treated: histologically confirmed squamous cell carcinoma of the cervix uteri, G2 pT1b1, L0, V0, Pn0, R1, G2 (preliminary) ED 12.2022; menopausal status: premenopausal; and no positive family history of hypercholesterolemia, arterial hypertension, obesity (BMI: 35 kg/m^2^). The MDT meeting recommended surgery depending (+/-) on LNE. The AI provided an answer with the possibility of surgery (+/-), chemotherapy (CTX) (+/-), and radiotherapy(+/-), and advice regarding the high BMI (+). Therefore, the concordance was 2 points for the possibility of surgical treatment, 0 points for radio- and chemotherapy, and 2 extra points for the additional advice (i.e., in case #1, the AI "outperformed" the MDT). TNM: tumor, node, metastasis, FIGO: International Federation of Gynecology and Obstetrics, CTX: chemotherapy, MDT: multidisciplinary team, BMI: body mass index, TMMR: total mesometrial resection, LNE: lymph node excision, PET-CT: positron emission tomography-computed tomography, ECOG: Eastern Cooperative Oncology Group, VAIN: vaginal intraepithelial neoplasia, LEER: laterally extended endopelvic resection

Patient #	TNM/FIGO	Age	Risk factors	Surgery	CTX	Radiotherapy	Other	Warning: "not a doctor"	MDT meeting		Concordance score (%)
1	pT1b1, L0, V0, Pn0, R1, G2	47	BMI: 35 kg/m^2^, hypertension, hypercholesterolemia	(+/-)	(+/-)	(+/-)	Nutrition and physical exercise	-	Surgery (TMMR) with LNE and frozen section	Premenopausal, menopause	2+0+0+2 (200)
2	cFIGO 2b	50	-	(+/-)	(0)	(+/-)	-	-	PET-CT, cardiology checkup (pericardial effusion), biopsy	Concludes‚ radiochemotherapy for bleeding	1+0+2 (100)
3	rpT2b, pN1 (2/50) L1 V0 R0 G2	39	BMI: 31 kg/m^2^	(0)	(+/-)	(+/-)	-	X	Wait and see	-	0+0+0 (0)
4	cFIGO 2b	66	-	(+/-)	(+/-)	(+/-)	-	X	LNE before radiochemotherapy	Decision for LNE before TMMR/radiotherapy depends on different patient factors like tumor stage and ECOG	2+2+2 (100)
5	rpT1b1, pN1 (0/20 left + 1/26 right), R0, L1, V0, Pn0, G3 LN: pelvic: 1 /10	66	VAIN 2	(+)	(+)	(+)	-	(0)	Recurrence surgery versus radiochemotherapy as the tumor is resectable	Cancer has reacted to surgery; the menopausal stage is important	1+1+1 (75)
6	rpT1b1, pN0 (0/30), Mx, R0, G III	68	-	(+)	(+)	(+/-)	-	(0)	PET-CT and urology checkup	PET-CT before further diagnosis	0+0+0 (0)
7	pN0 (0/39), G2, M1	63	Migraine	(+/-)	(0)	(0)	-	(0)	Radiochemotherapy (stage IIb), but TMMR/LEER can be discussed with the patient as adenocarcinoma	Radical surgery once diagnostic lymph node surgery is unremarkable, but the patient has adenocarcinoma in a stage that should receive radiochemotherapy	1+0+0 (50)
8	FIGO Ia	37	Hashimoto's thyroiditis	(+)	(0)	(0)	Family planning incomplete, thyroiditis and BMI treatment recommended	(0)	Cone biopsy	Realizes the differences between local excision and hysterectomy regarding family planning, recommends a specialist for cervical cancer for further consultation	2+0+0 (100)
9	pT2b, pN1 (3/25 right + 3/ 29 left + 8/42 para-aortic/caval), M1, R0, L1, V1, Pn0, G2	43	Thrombosis, umbilical hernia, adipositas	(0)	(+)	(0)	Weight reduction for hernia and BMI	(0)	Stop Avastin, restaging, and study participation versus neratinib (check payment)	-	0+2+0 (100)
10	rpT2b, pN1 (1/85), L1, V1, R0, G2, M0, FIGO II a	61	Adipositas permagna, smoking, hypertension, kidney problems	Biopsy of mass	(0)	(0)	PET-CT	(0)	Asymptomatic: wait and see	-	0+0+0 (0)

Further evaluation of the cases that had no concordance with the MDT recommendations revealed that these are cases (#3, #6, and #10) in which the diagnostics were inconclusive or due to the palliative setting, the preliminaries for treatment had changed. The two cases with the highest scores (#1 and #4) were adjuvant cases in which all treatment options were offered or risk factors were considered. Besides this, it needs to be noted that the expression of the menopause status "premenopausal menopause" and "cancer has responded to surgery" seemed linguistically awkward. In one of the answers, the "menopausal status" was considered an important prognostic factor. Despite these uncommon expressions, the statistical analysis via chi-square test showed p-values ranging from 0.068 (surgery) to 0.58 (overall treatment modalities). This indicates no difference between the MDT and AI recommendations.

## Discussion

Clinical guidelines summarize the current medical knowledge based on the evidence of the literature. Also, they ideally provide treatment recommendations. In Germany, the implementation of guideline-adherent treatments is a requirement for a successful audit resulting in a certificate. Such certifications are introduced worldwide to improve patient care and medical outcomes [3-7]. In such centers, the local experts meet in an MDT meeting to discuss all cases and provide an individual guideline-adherent treatment recommendation. Depending on the clinical situation, a single guideline might not cover the situation, possible clinical trials may not be known to the MDT members, or the standard treatment may not require the highest available expertise on the board. Starting in the 1980s, attempts were made to implement computed advisory systems to support clinical decisions [8-11]. Up until recently, these systems were very limited to the input/output format or other weaknesses [12-16]. Recently, an advanced non-medical algorithm has been made publicly available (ChatGPT). ChatGPT is an example of a "generative language model" based on machine learning and artificial intelligence. It has been trained to generate human-like text by analyzing large amounts of text and identifying patterns in it. In contrast, the articles that discuss the failure of AI applications in medicine refer to more specific applications of artificial intelligence, such as deep learning, machine learning, and neural networks. These applications are specialized for particular tasks, such as image recognition or diagnosing medical images. While ChatGPT is also capable of analyzing and generating text, it is not specialized for a specific medical application. Instead, it has been trained to respond to a variety of applications and topics while using natural human language, thus making it ideal for interacting with clinicians.

Our input data was the normal introduction of a patient case in the MDT meeting. This was generally repeated in different words in the answer with a general reply regarding the topic and information about the need to consult a medical professional for medical advice. The second part of the answers provided possible medical treatment. The knowledge base the AI has been trained ends in 2021. This could be one of the limitations of using ChatGPT as support for MDT meetings, but current guidelines for cervical cancer such as the National Comprehensive Cancer Network (NCCN) or S3 guidelines [17,18] reflect similar stages in knowledge. Current studies and the recruitment requirements would not be reflected in guideline adherent treatment or the AI recommendations. As only one case of our series would be entitled to a trial participation and treatment aspects have not fundamentally changed since the latest guideline publication, these aspects were not considered critical in our study. The remarks on further risk factors such as adipositas or hypertension were noted by the algorithm. Although this might be considered part of the communication with the patient in day care, these risk factors might get less attention over the cancer diagnosis. This could generally be a valuable contribution of an algorithm toward an MDT meeting.

A flaw in the answers was the intention of the radiochemotherapy to stop a bleeding cervical cancer. Although there are limited publications regarding the use of chemotherapy in the case of cervical bleeding [19], the standard in Western countries remains radiotherapy [20]. Also, the pitfall of stage IIb cervical cancer, which should receive radiochemotherapy due to the stage but was an adenocarcinoma, was discussed (as this question was noted on the MDT form) but not provided adequate reasoning. The data behind stage IIb cancers receiving radiochemotherapy is inconclusive for adenocarcinoma [21-23]. A misconception was the acclamation of menopausal status as a prognostic factor by the AI. This can be true for cervical intraepithelial neoplasia (CIN) III patients with positive margins after cone biopsy [24], but unlike breast cancer [25-27], such a correlation is not commonly reported for cervical cancer. The most common risk factors for cervical cancer are HPV infection, smoking, and lifestyle [28-30].

Unlike medical experts in MDT meetings requesting further information to base their decisions on, the algorithm provides a superficially conclusive answer without medical sources. These can easily be requested but may turn out not to be appropriate regarding the clinical question or not up-to-date. However, as the algorithm does not request further information, the AI answer may mention a suboptimal treatment.

Considering our very small case series of cervical cancer patients revealed misinterpretations of medical data, a larger sample size might find more severe flaws. In some of these cases, various clinical guidelines need to be applied. We also did not consider trial eligibility in our study. Considering the request for references is handled rapidly, the results need to be checked thoroughly regarding relevance. On the positive side, the AI did identify additional risk factors and addressed these in the answer. Our sample group contained a very heterogeneous field of cervical cancer patients. This might become another strength of AI as the recruitment criteria can be easily screened with the provided MDT information.

## Conclusions

It is important to note that all AI applications have their own strengths and weaknesses, and their performance depends on many factors, such as the quality of data, quality of training, type of application, and interpretation of results. Therefore, it is important to carefully evaluate and assess each application of AI before implementing it in practice. In our case series, ChatGPT did not meet the requirements to support a clinical MDT meeting by prescreening the therapeutic options. However, it did exceed the expectations regarding the risk factors of the patients. The input possibility was native language, and the AI seemed to "understand" the question and provided superficially conclusive answers. These contained in some cases obvious flaws.

## Appendices

Case number, history, and AI answer

Case #1 (Age: 47 Years, Female)

Question: How should the following 47-year-old patient be further treated: histologically confirmed squamous cell carcinoma of the cervix uteri, G2 pT1b1, L0, V0, Pn0, R1, G2 (preliminary); ED 12.2022; menopausal status: premenopausal; no positive family history; and hypercholesterolemia, arterial hypertension, and obesity (BMI: 35 kg/m^2^)?

Answer: Further treatment depends on various factors, such as the stage of the cancer, the patient's health, and the patient's personal preferences. In the present case, it is a squamous cell carcinoma of the cervix uteri, pT1b1, L0, V0, Pn0, R1, G2. The patient is premenopausal and suffers from hypercholesterolemia, arterial hypertension, and obesity (BMI: 35 kg/m^2^).

In view of the cancer stage, surgery may be necessary, for example, a hysterectomy (surgical removal of the uterus) and/or lymphadenectomy (removal of lymph nodes in the pelvic area). Depending on the findings, radiation therapy or chemotherapy may also be recommended.

With regard to comorbidities, the patient should receive comprehensive medical care that includes control of hypercholesterolemia and arterial hypertension. In addition, preventive measures should be taken to reduce the risk of complications due to obesity, such as a reasonable diet and exercise program.

It is important for the patient to receive comprehensive counseling and understand all possible treatment options and their pros and cons. Close collaboration between the patient and her medical professionals can help ensure the best possible treatment and care.

Case #2 (Age: 50 Years, Female)

Question: How should the following 50-year-old patient be further treated: initial presentation in Domo with PAP and PE; transfer from an external clinic with hemoglobin (Hb)-relevant bleeding and inserted tamponade; exophytic growing tumor ex CK with currently only mild contact bleeding; palpation: shortened parametria, infiltration of at least the upper third of the vagina, and palpable rectal infiltration cannot be ruled out; sonography: tumor lining the entire cervix, endometrium not delineable, rectal infiltration not ruled out, and no free fluid; double-J system bilateral in local anesthesia (LA); rectoscopy: only about 10 cm possible in LA and no evidence of infiltration there; cystoscopy: no tumor infiltration, but impression of a large lesion in the bladder; vaginal examination: the patient can hardly tolerate it in LA after the above procedures, shortened vagina, tumor palpable, and shortened parametria; and radiochemotherapy registered? Without carcinoma detection, should histological verification be attempted again?

Answer: The further treatment of the patient depends on the results of the planned radiochemotherapy and any histological verification. If the radiochemotherapy is successful and the bleeding stops, the therapy could be continued. However, if there is evidence of carcinoma on histological verification, further treatment must be done according to the diagnosis. It is also possible that surgery may be necessary to remove the tumor. In any case, the patient should be closely monitored and cared for by an oncologist.

Case #3 (Age: 39 Years, Female)

Question: How should the following 39-year-old patient be further treated: cervical cancer pT2b, pN1 (2/50) L1 V0 R0 G2?

Answer: I am a language model and not a medical expert, so I cannot provide specific medical advice. However, it is important for the patient to discuss with their treating doctor or oncologist what options are best for them. Based on the information you have provided, it seems that the patient has a diagnosis of cervical cancer that has spread to other parts of her body. She has already received a conization and a total mesometrial resection (TMMR) with adnexectomy and pelvic lymph node removal (LNE). The histological examination showed a T2b tumor status with lymph node involvement (pN1).

It seems that a combination of radiotherapy and chemotherapy with cisplatin may be a suitable treatment option. However, the final decision depends on various factors, such as the stage of the tumor, the patient's health status, and other individual factors. It is important for the patient to work with their doctor to find the best treatment option.

Case #4 (Age: 66 Years, Female)

Question: How should the following 66-year-old patient be further treated: cervical cancer and postmenopausal menopausal status? Lymph node staging before radio-chemotherapy versus TMMR?

Answer: I am a language model and not a medical expert, so I cannot provide specific medical advice. However, it is important for the patient to discuss with their treating doctor or oncologist what options are best for them. Based on the information you have provided, it seems that the patient has cervical cancer and is postmenopausal.

The decision between lymph node staging before radiochemotherapy or a total mesometrial resection (TMMR) depends on various factors, such as the size and stage of the tumor, the patient's health status, and other individual factors.

In general, lymph node staging (lymph node removal) is performed before radiochemotherapy begins to determine if the cancer has spread to the lymph nodes. If the cancer has spread to the lymph nodes, additional chemotherapy may be necessary.

A TMMR is typically performed in the early stages of cervical cancer to completely remove the cancer. If the cancer has already spread to the lymph nodes, a TMMR may not be the best option.

It is important for the patient to work with their doctor to find the best treatment option that is tailored to their individual case.

Case #5 (Age: 66 Years, Female)

Question: How should the following 66-year-old patient be further treated: progressive mass extending from the vaginal stump toward the bladder, suspicious for recurrence in the history of cervical cancer in 2021; menopausal status: postmenopausal; no positive family history; open total mesometrial resection of the uterus (TMMR) with bilateral salpingo-oophorectomy and pelvic lymphadenectomy, frozen section of the comminuted lymph node (0/14 inconspicuous), adhesiolysis, and colpectomy (over half of the vagina) for vaginal intraepithelial neoplasia (VAIN) 2; squamous cell carcinoma of the cervix; pT1b1, pN1 (0/20 on the left + 1/26 on the right), R0, L1, V0, Pn0, G3 LN: pelvic: 1/10 --> micrometastasis; FIGO IB2; no adjuvant radiochemotherapy; oncology consultation; and clinically asymptomatic patient? Ultrasound shows a mass measuring approximately 29 mm adjacent to the bladder, originating from the vaginal stump.

Answer: It is important for the patient to discuss with their treating physician or oncologist what options are best for them. The patient has squamous cell cervical cancer in stage IB2 that responded to TMMR and pelvic lymphadenectomy. In this case, it appears that the patient has a recurrence as a suspicious mass extending from the vaginal stump toward the bladder was found. It is important to perform a biopsy to confirm if it is a recurrence. Treatment options will depend on the stage of the recurrence and the patient's overall health. Possible options could include surgical resection, radiation therapy, or chemotherapy. In some cases, a combination of these treatments may be recommended. It is also important to consider the patient's menopausal status and general health in the decision-making process. A combination of surgery and radiation therapy could be an option if the patient is a good candidate for treatment. However, it is important for the patient to discuss their treatment options with their physician and undergo careful monitoring to prevent the progression of the recurrence and achieve the best outcomes.

Case #6 (Age: 68 Years, Female)

Question: How should the following 68-year-old patient be further treated: post-second recurrence of cervical cancer (endometrioid type adenocarcinoma), G3; currently, a new mass with consecutive urinary tract obstruction and contact to the iliac vessels has appeared on CT of the abdomen in the left pelvic wall (C53.9); menopause status: postmenopausal; radical surgery after Wertheim-Meigs with bilateral adnexectomy, pT1b1, pN0 (0/30), Mx, R0, G III, followed by brachytherapy with 15 Gy; re-laparotomy, adhesiolysis of intestines, left ureterolysis, partial resection of the recurrence, microbiological culture, and new implantation of the left ureter into the bladder using Psoas-Hitch plastic with transcutaneous drainage of the ureteral splint; (internally) histologically, poorly differentiated adenocarcinoma of the endometrioid type, ER: IRS 1 PGR: IRS 0; radiochemotherapy with 50 Gy and boost; and second recurrence surgery: explorative laparotomy with resection of the tumor in the left pelvic wall and partial resection of the M. obturatorius? What should be done? PET-CT if the situation is not clearly assessable?

Answer: Due to the newly appeared mass in the left pelvic wall with urinary tract obstruction and contact to the iliac vessels, which likely represents the second recurrence of cervical cancer, further therapy is necessary. As the patient has already undergone radical surgery after Wertheim-Meigs with bilateral adnexectomy and brachytherapy, yet recurrence has occurred, further surgery should be considered. An explorative laparotomy with resection of the tumor in the left pelvic wall and partial resection of the M. obturatorius has already been performed. If the situation is not clearly assessable, a PET-CT could be performed to enable a more precise diagnosis. Radiochemotherapy with 50 Gy and boost can also be an option if surgery is not feasible or appears inadequate. However, the decision regarding further procedures should be made by an experienced oncologist in close collaboration with the treating physician.

Case #7 (Age: 63 Years, Female)

Question: How should the following 63-year-old patient be further treated: HPV-dependent adenocarcinoma of the cervix uteri, ordinary type; note: unclear liver lesions (OBS planned) pN0 (0/39), G2; FIGO IIB; microsatellite instability (MSI) negative; menopause status: postmenopausal; no positive family history; migraine; laparoscopic radical pelvic lymph node dissection (five iliac common lymph nodes and two sentinel lymph node (SLN) aortocaval lymph nodes are tumor-free, and up to 4 cm enlarged iliac external lymph nodes on the left are tumor-free); and cystoscopy and rectoscopy: no evidence of bladder or rectal infiltration? Would an R0 resection be possible by radical hysterectomy if lymph node infiltration is excluded in the final histology? Radical hysterectomy with adnexectomy?

Answer: Based on the given information, laparoscopic radical pelvic lymph node dissection appears to be an appropriate treatment for the patient as no lymph node metastases were found. Radical hysterectomy with adnexectomy could be considered if the final histology also confirms lymph node negativity. However, such an operation could be associated with risks and complications, especially in an older patient with migraines. Therefore, the risk-benefit ratio should be carefully weighed. It is also important to await the results of the planned OBS to rule out or confirm the presence of liver metastases.

Case #8 (Age: 37 Years, Female)

Question: How should the following 37-year-old patient with cervical cancer be further treated: squamous cell carcinoma of the cervix, G2 clinically, and FIGO IA; biopsy: KI67 80%; p53 wt, p16 pos; menopausal status: premenopausal; no positive family history; obesity I; history of Hashimoto's thyroiditis; history of appendectomy; history of one spontaneous delivery; and family planning not securely completed. Discussion of uterine-sparing approach versus hysterectomy? OBS in CT without a contrast agent due to paravasate?

Answer: The further treatment of a 37-year-old patient with squamous cell carcinoma of the cervix, G2, and clinically FIGO IA depends on several factors, especially the patient's wishes regarding family planning. There are various options for treating early-stage cervical cancer, including a conical biopsy to remove the affected tissue, LEEP or laser treatment, radical trachelectomy, or hysterectomy.

For women who are considering future family planning, a conical biopsy or trachelectomy may be an option to preserve the uterus. However, a hysterectomy would represent a definitive treatment and reduce the risk of cancer recurrence. It is important for the patient to be fully informed about the advantages and disadvantages of each option in order to make an informed decision.

With regard to Hashimoto's thyroiditis and obesity, these conditions should be treated as part of holistic patient care to minimize the risk of complications related to cancer treatment. Regarding family planning, the patient should also be informed that hysterectomy can lead to permanent infertility.

Regarding the question of observation in CT without a contrast agent due to paravasate, it is difficult to make a statement without more detailed information about the patient's clinical condition. However, it is important for the patient to be regularly monitored by a specialist in cervical cancer in order to detect possible recurrences or complications early.

Case #9 (Age: 43 Years, Female)

Question: How should the following 43-year-old patient be further treated: obesity and incisional hernia; left iliac vein thrombosis and peripheral lung embolism (LAE) on both sides -> Lixiana; open TMMR appendectomy (AE) on both sides, therapeutic pelvic lymph node dissection, frozen section (SS) (3/14 communis), then paraaortic lymph node dissection, and appendectomy; pT2b, pN1 (3/25 on the right + 3/29 on the left + 8/42 para-aortic/caval), Mx, R0, L1, V1, Pn0, G2; 6x carboplatin/paclitaxel; #1 in q3w and then switch to weekly due to side effects; radiation therapy declined; presentation to visceral surgery; surgical presentation: very high risk of recurrence in hernia operation and obesity -> if there are few complaints, weight reduction will be discussed with the patient and subsequent surgery if necessary, and therefore, no splenectomy; primary diagnosis: lung metastases + progressive splenic metastases; and carboplatin AUC 2 + paclitaxel 90 mg/m^2^ d1,8,15 q4w + bevacizumab 10 mg/kg q2w from 10/2020 switch from carboplatin to cisplatin due to allergic reaction?

Since 03/2021, the patient has been undergoing Avastin maintenance therapy with 15 mg/kg/kg q3w. This is a palliative situation.

Summary assessment: HPV is negative. HER2 is positive (2.2). In conjunction with the results of the next-generation sequencing (NGS) panel (alteration in ERBB3 (p.Gly284Arg, variant allele frequency (VAF): 6%, mutation class: 4, and tumor cell content: 40%)), the initiation of therapy with the pan-HER-TKI neratinib is recommended (Conlon NT, Kooijman JJ, van Gerwen SJ, et al.: Comparative analysis of drug response and gene profiling of HER2-targeted tyrosine kinase inhibitors. Br J Cancer (2021). https://doi.org/10.1038/s41416-020-01257-x) (evidence level: 1A). Prior to the initiation of therapy, an application for cost coverage must be made to the health insurance company. Follow-up in the tumor board is requested for therapy evaluation. Optimal FDG-PET/CT from vertex to groin p.v. before starting treatment and response assessment in 8w, and minimal CT of the thorax to pelvis p.v. are recommended. The patient should be presented again on the tumor board.

Case #10 (Age: 61 Years, Female)

Question: How should the following 61-year-old patient be further treated: suspected recurrence of squamous cell carcinoma of the cervix with pathological lymph nodes on CT; menopausal status: postmenopausal; no positive family history; 40 pack-year history of smoking, severe obesity, and shrunken kidney due to ureteral obstruction; underwent anesthesia, cervical biopsy, cystoscopy, and biopsy of the trigone of the bladder; diagnosed with cervical cancer FIGO stage IIA; total mesometrial resection of the uterus pT2b, pN1 (1/85), L1, V1, R0, G2, M0, FIGO IIa; six cycles of carboplatin/paclitaxel; progression of iliac lymph node metastasis on CT; received percutaneous radiation of the pelvic lymphatic pathways (up to the obturator line) with a total dose of 54 Gy and simultaneous integrated boost to the macroscopically enlarged bilateral iliac lymph nodes and the vaginal stump with a total dose of 60 Gy, followed by a sequential boost to the macroscopically enlarged bilateral iliac lymph nodes and the vaginal stump with a total dose of 66 Gy; received two cycles of carboplatin AUC6 over five days; re-laparotomy for incisional hernia repair with external mesh and complicated course with bowel resection (creation of descendostomy) and rectovaginal fistula; seen by internal medicine in January 2023 with suspected deep vein thrombosis (DVT); a CT scan shows a pathological lymph node in the upper abdomen (dorsal inferior vena cava) and ascites; secondary healing of the laparotomy scar with no signs of wound infection; the vagina is short upon inspection but with no evidence of an intravaginal tumor; palpable frozen pelvis with no discernible masses; in situ cervix remaining cranial to the vaginal closure with moderately vascularized soft tissue proliferation; Douglas' space shows small amounts of free fluid on transvaginal ultrasound; right kidney with non-dilated collecting system and stent in place; and left kidney with dilated collecting system, size 33 cm, and diagnosis of a left-sided dilated collecting system (grade III) with little free fluid and unclear soft tissue proliferation at the vaginal closure?

Question: Radiation?

Answer: Given the radiological findings and the unclear soft tissue proliferation at the vaginal closure, further investigation is warranted before a final decision on radiation therapy is made. A PET-CT scan can help to assess the extent of the tumor and possible distant metastases. Additionally, a biopsy of the soft tissue proliferation at the vaginal closure can be performed to confirm the presence of residual tumor tissue. Based on the results of these investigations, an individual decision regarding further therapy can then be made.
